# Clinical Outcome and Risk Factors for Progression of Prenatally Diagnosed Fetal Ventriculomegaly: A Retrospective Multicenter Study

**DOI:** 10.1002/pd.6816

**Published:** 2025-05-19

**Authors:** Anouk Moens, Zoe Albersnagel, Marieke B. Veenhof, Phebe N. Adama van Scheltema, Esther Sikkel, Mariëtte J. V. Hoffer, Brigitte H. W. Faas, Dineke Westra, Ilse Feenstra, Emilia K. Bijlsma, Gijs W. E. Santen, Corrie E. Erasmus, Cacha M. P. C. D. Peeters‐Scholte

**Affiliations:** ^1^ Department of Neurology Leiden University Medical Center Leiden the Netherlands; ^2^ Department of Obstetrics and Gynecology Division of Fetal Medicine Leiden University Medical Center Leiden the Netherlands; ^3^ Department of Obstetrics and Gynecology Radboud University Medical Center Nijmegen the Netherlands; ^4^ Department of Pediatric Neurology Radboud University Medical Center Amalia Children's Hospital Nijmegen the Netherlands; ^5^ Department of Clinical Genetics Leiden University Medical Center Leiden the Netherlands; ^6^ Department of Genetics Radboud University Medical Center Nijmegen the Netherlands

**Keywords:** exome sequencing‐neurosonography, fetal counseling, fetal ventriculomegaly, hydrocephaly, outcome, termination of pregnancy

## Abstract

**Objective:**

To investigate the clinical outcome of fetuses with ventriculomegaly (VM), and to identify risk factors for progression of fetal VM in order to improve prenatal counseling. This was a multicenter, retrospective cohort study, comprising 229 cases with VM.

**Methods:**

VM was classified as mild, moderate, or severe and isolated or non‐isolated. Genetic data were collected. Differences between VM subgroups were described, and risk factors for progression of fetal VM were identified using logistic regression analysis. Outcome was defined as the percentage of live births, termination of pregnancy (TOP) and intra‐uterine fetal demise (IUFD).

**Results:**

Of the 229 cases, 109 (47.6%) had mild VM, 60 (26.2%) moderate VM, and 60 (26.2%) severe VM. Progression of VM occurred in 45/153 cases (29.4%), half of which were in the group with severe VM. Dilatation of the 3rd ventricle and neural tube defects were risk factors for progression of VM. The percentage of live births (excluding cases with TOP and unknown outcome) was 93.1% (54/58) in mild VM, 78.6% (22/28) in moderate VM and 92.6% (25/27) in severe VM. In 12/229 cases (5.2%) IUFD occurred. Genetic analysis was performed in 143/229 (62.4%) of cases, showing (likely) pathogenic abnormalities in 41/143 (28.7%) cases, predominantly in mild, non‐isolated VM.

**Conclusions:**

This study confirms the clinical relevance of additional genetic investigations in all types of fetal VMs. Further larger prospective research including clinical follow‐up is needed to improve prenatal counseling.

**Summary**

- What's already known about this topic?
  - Fetal ventriculomegaly is one of the most common fetal anomalies.
  - Outcome is dependent on the severity of the ventriculomegaly and the presence of associated structural abnormalities.
- What does this study add?
  - Progression of fetal ventriculomegaly occurred in about 30% of cases, especially in severe ventriculomegaly.
  - Risk factors for progression of ventriculomegaly are presence of 3rd ventricular dilatation and neural tube defects.
  - Additional genetic evaluation is advisable in all types fetal of ventriculomegaly.

## 1 Introduction

Fetal ventriculomegaly (VM) is one of the most frequently occurring anomalies in pregnancy with an estimated incidence of 0.3–1.5 per 1000 pregnancies [1]. It is defined as an atrial diameter of the lateral ventricle of 10 mm or more on ultrasound, regardless of the gestational age. VM can be subdivided into mild (10–11.9 mm), moderate (12–14.9 mm), and severe (≥ 15 mm) and can be either isolated or non‐isolated/complex when other (extra) central nervous system anomalies are found [2].

In the Netherlands, when fetal VM is detected on ultrasound, parents are referred to a tertiary hospital and counseled regarding the potential risks and outcome for the fetus. The prognosis of the fetus depends on several factors, such as the presence of associated anomalies, uni‐ or bilateral involvement of the cerebral ventricles, the ventricle size and the progression of the VM over time, and/or the presence of an underlying genetic disorder or infection. Due to the numerous contributing factors, prenatal counseling of parents with a fetus diagnosed with VM is very challenging [1]. Furthermore, outcome has to be predicted at an early stage of pregnancy as many countries have a legal limit of termination of pregnancy (TOP). Recently, Single Nucleotide Variant (SNV) analysis via exome sequencing (ES) has been introduced in the counseling of fetuses with anomalies on fetal ultrasound [3]. In a recent meta‐analysis, a high diagnostic yield was demonstrated in fetuses with severe bilateral VM after negative chromosomal micro‐array (CMA) [4]. The aim of this study was to predict the outcome of fetuses with prenatally diagnosed VM and to identify risk factors for abnormal outcome in order to improve prenatal counseling.

## 2 Methods

### 2.1 Study Design and Population

**FIGURE 1:**
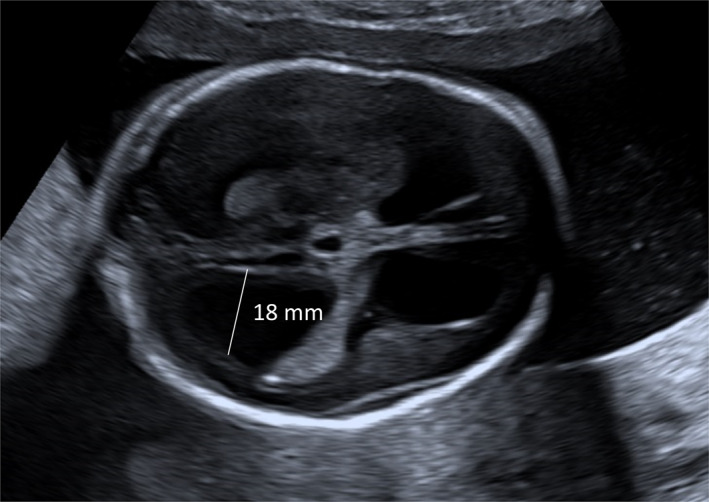
Ultrasound at 19 weeks of gestation, showing severe ventriculomegaly.

This study is a retrospective cohort study of all patients with fetal VM diagnosed by ultrasound from January 01, 2015 to September 01, 2020 at the Leiden University Medical Center and Radboud University Medical Center. VM was categorized into mild, moderate, or severe based on the measurements on the first prenatal ultrasound performed at one of both medical centers (Figure 1). Furthermore, fetal VM cases were subdivided into isolated (no other (extra)cranial abnormalities) or non‐isolated (with (extra)cranial abnormalities). No exclusion criteria were used. The Medical Ethics Committees of the Leiden and Radboud University Medical Centers issued a waiver of approval. Since only retrospectively collected, clinically pseudonymous data were used, no parental or guardian consent was required, in accordance with Dutch guidelines.

### 2.2 Data Collection

#### 2.2.1 Perinatal Data

In the Netherlands, all pregnant women are offered a structural antenatal ultrasound at 18–20 weeks of gestation. Any abnormal findings detected during these screenings prompt referral to tertiary hospitals for additional diagnosis and counseling. All cases with fetal VM referred to either Leiden University Medical Center or Radboud University Medical Center were included in this study. Demographics such as maternal age, gravida, parity, miscarriages and gestational age at diagnosis were described. Data about clinical characteristics and possible risk factors for VM, such as additional anomalies on ultrasound, unilateral or bilateral VM, and fetal gender, were also collected. [2, 5] Cases with fetal VM diagnosed at a later gestational age were also included.

#### 2.2.2 Imaging

During the antenatal ultrasound, the atrium of the ventricle was measured in the transventricular‐axial plane at the level of the parieto‐occipital sulcus. VM was subdivided into mild (10.0–11.9 mm), moderate (12.0–14.9 mm), and severe VM (≥ 15 mm). Also, the presence of unilateral versus bilateral VM and involvement of the 3rd and 4th ventricles were scored. Unilateral VM was defined as one lateral ventricle with an atrial measurement of 10 mm or more [6], while asymmetric VM was defined as a difference of ≥ 2 mm between both enlarged lateral ventricles [7]. Progression or regression of VM was defined as an increase or decrease of atrial width of ≥ 3 mm between the first and last ultrasound, respectively. In some cases, a fetal MRI was performed to establish or confirm a diagnosis. Data of these MRI findings were collected and additional radiological abnormalities were classified into central nervous system and non‐central nervous system anomalies.

#### 2.2.3 Genetics and Laboratory Data

If available, data from non‐invasive prenatal testing were collected. All women were offered amniocentesis for additional fetal genetic evaluation by QF‐PCR to screen for the most commonly occurring aneuploidies (trisomies 13, 18, and 21 and sex chromosomal aneuploidies), CMA and/or SNV analysis via ES. There were no patient‐related costs for genetic testing as all costs of testing were covered by the public health system. Data of these genetic tests were collected, as well as data of maternal infectious serology (such as Toxoplasmosis, Rubella virus, Cytomegalovirus, Herpes simplex virus, and Treponema pallidum). Data on the presence of fetal or neonatal alloimmune thrombocytopenia were also obtained. Maternal infectious serology and thrombocyte antibody testing were only offered in case there was suspicion of either infection of bleeding, respectively.

#### 2.2.4 Outcome

Outcome parameters were the percentages of live births, TOP, and intra‐uterine fetal demise (IUFD). Pregnancies with unknown outcomes were not included in the percentage of live births. Predefined risk factors for progression, stability or regression of VM during pregnancy were fetal gender, singleton or twin pregnancy, change in VM diameter during pregnancy (regression, stable or progression), dilatation of the third, and/or fourth ventricle, uni‐ or bilateral VM, isolated, or non‐isolated VM (subdivided by the presence of additional CNS‐ or non‐CNS malformations), and the presence of genetic abnormalities as detected by QF‐PCR, CMA and/or ES.

#### 2.2.5 Statistical Analysis

Descriptive statistics were used to analyze demographic data and clinical characteristics in the three defined VM subgroups. Variance homogeneity was assessed using the Levene's test. Means with standard deviations were calculated for continuous normalized variables and medians with interquartile range for not‐normalized data. Percentages were calculated for non‐continuous variables. One‐way ANOVA with post hoc analysis (Bonferroni) was performed to identify differences between VM subcategories. Missing data were not imputed.

A backward logistic regression model was constructed to identify independent prognostic factors for progression of fetal VM. For this analysis, only cases for which information about the progression status (no progression, defined as resolved, regressed and stable VM vs. progression) was available were eligible. Cases with missing values in any of the variables included in the backward selection process were excluded from the analysis. First, univariable regression analyses were performed, in which all possible risk factors were evaluated. Variables with a *p*‐value < 0.200, that were clinical relevant and consistent with previous literature, were eligible for inclusion in the multivariable analysis, which was used to evaluate which factors were independently associated with progression of VM (defined as a *p*‐value > 0.05). Stopping criteria for the backward selection process were based on statistical significance (*p* < 0.05), meaning variables with *p* ≥ 0.05 were removed sequentially. Statistical analysis was performed with IBM SPSS Statistics (version 23.0, New York, NY, United States 2020).

## 3 Results

### 3.1 Demographics and Clinical Characteristics

During the study period, data from 229 cases were extracted from the patient data system of the two sites. Of all VM cases, 109/229 cases (47.6%) were classified as mild, 60/229 cases (26.2%) as moderate, and 60/229 cases (26.2%) as severe. Maternal and fetal characteristics at diagnosis are described in Table 1. No significant differences in maternal characteristics were identified between the VM categories. Regarding fetal characteristics, isolated and unilateral VM were more prevalent in mild VM than in moderate and severe VM. Dilatation of the 3rd and 4th ventricles was most prevalent in severe VMs.

**TABLE 1:** Demographics and clinical characteristics at diagnosis.

	Mild	Moderate	Severe	Total
	*n* = 109	*n* = 60	*n* = 60	*n* = 229
Maternal characteristics				
Maternal age, mean in years (SD)	30.86 (5.20)	31.50 (5.10)	30.88 (4.85)	
Gestational age at diagnosis				
< 24 weeks	78 (71.6%)	44 (73.3%)	37 (61.7%)	*n* = 159
≥ 24 weeks	31 (28.4%)	16 (26.7%)	23 (38.3%)	*n* = 70
Parity				
0	49 (45.0%)	19 (31.7%)	28 (46.7%)	*n* = 96
1	34 (31.2%)	27 (45.0%)	22 (36.7%)	*n* = 83
2	18 (16.5%)	8 (13.3%)	7 (11.7%)	*n* = 33
≥ 3	8 (7.3%)	7 (11.7%)	2 (3.3%)	*n* = 17
Miscarriages				
0	74 (67.9%)	35 (58.3%)	42 (70.0%)	*n* = 151
1	19 (17.4%)	19 (31.7%)	11 (18.3%)	*n* = 49
2	12 (11.0%)	4 (6.7%)	6 (10.0%)	*n* = 22
≥ 3	4 (3.7%)	2 (3.3%)	1 (1.7%)	*n* = 7
Type of pregnancy				
Singleton	101 (92.7%)	51 (85.0%)	56 (93.3%)	*n* = 208
Twin	8 (7.3%)	9 (15.0%)	4 (6.7%)	*n* = 21
Mode of conception				
Spontaneous	56 (51.3%)	36 (60.0%)	41 (68.3%)	*n* = 133
Fertility treatment	7 (6.4%)	9 (15.0%)	6 (10.0%)	*n* = 22
Unknown	46 (42.2%)	15 (25.0%)	13 (21.7%)	*n* = 74
Consanguinity				
Yes	5 (4.6%)	3 (5.0%)	2 (3.3%)	*n* = 10
No	55 (50.4%)	31 (51.7%)	37 (61.7%)	*n* = 123
Unknown	49 (45.0%)	26 (43.3%)	21 (35.0%)	*n* = 96
Fetal Characteristics				
Fetal gender				
Male	62 (52.1%)	32 (53.3%)	27 (45.0%)	*n* = 121
Female	45 (41.2%)	27 (45.0%)	33 (55.0%)	*n* = 105
Unknown	2 (1.7%)	1 (1.7%)	0	*n* = 3
Isolated VM	52 (47.7%)	16 (26.7%)^d^	8 (13.3%)^b^	*n* = 76^a^
Non‐isolated VM	57 (52.3%)	44 (73.3%)	52 (86.7%)	*n* = 153
Unilateral VM	36 (33.0%)	8 (13.3%)^d^	6 (10.0%)^b^	*n* = 50^a^
Bilateral VM	73 (67.0%)	52 (86.7%)	54 (90.0%)	*n* = 179
Symmetrical	72 (65.1%)	50 (83.3%)	35 (58.3%)^b^	*n* = 157^a^
Asymmetrical	1 (0.9%)	2 (3.3%)	19 (31.7%)	*n* = 22
Dilatation of the third ventricle	10 (9.2%)	17 (28.3%)^d^	26 (43.3%)^b^	*n* = 53^a^
Dilatation of the fourth ventricle	1 (0.1%)	0^c^	7 (11.7%)^b^	*n* = 8^a^

Abbreviation: VM = ventriculomegaly.

^a^ One way Anova *p* < 0.01.

^b^ Post‐hoc analysis with Bonferroni correction: severe versus mild VM *p* < 0.01.

^c^ Moderate versus severe VM *p* < 0.01.

^d^ Moderate versus mild VM *p* < 0.05.

In total, 76/229 cases (33.2%) were defined as isolated VM, showing no other anomalies at the first advanced ultrasound or fetal MRI. In isolated VM, there were significantly more cases with mild VM (52/76; 68.4%) than with moderate (16/76; 21.1%) or severe VM (8/76; 9.5%; *p* < 0.01). In the non‐isolated VM group (153 cases; 66.8%), 4/153 cases (1.8%) had a proven infection during pregnancy, and 7/153 cases (3.1%) involved a cerebral bleeding. The additional abnormalities found on fetal ultrasound were categorized into central nervous system (CNS) malformations and non‐CNS malformations such as anomalies in the heart, face, skull, and/or extremities (Table 2). Additional CNS malformations (103/229 cases; 45.0% of all VM) were equally distributed among all VM subgroups. Aqueductal stenosis was significantly more common in severe VM as compared to moderate and mild VM (*p* < 0.01). Additional non‐CNS malformations were identified in 85/229 cases (37.1%), and were equally distributed over VM subgroups as well.

**TABLE 2:** Frequencies of isolated and non‐isolated anomalies of fetal ventriculomegaly.

	Mild VM	Moderate VM	Severe VM	Total
	*n* = 109 (47.6%)	*n* = 60 (26.2%)	*n* = 60 (26.2%)	*n* = 229 (100%)
Isolated	*n* = 52 (68.4%)	*n* = 16 (21.1%)^e^	*n* = 8 (9.5%)^b^	*n* = 76 (33.2%)^a^
Non‐isolated	*n* = 57 (37.3%)	*n* = 44 (28.8%)	*n* = 52 (34.0%)	*n* = 153 (66.8%)
CNS‐malformations	*n* = 32 (31.1%)	*n* = 30 (29.1%)^c^	*n* = 41 (39.8%)^b^	*n* = 103 (45.0%)^a^
Neural tube defect	14	11	8	*n* = 33
Holoprosencephaly	2	2	4	*n* = 8
Corpus callosum abnormality	7	5	12	*n* = 24
Aqueductal stenosis	0	2^d^	9^b^	*n* = 11^a^
Dandy–Walker continuum	4	4	5	*n* = 13
Cortical abnormality	3	2	3	*n* = 8
Cerebellar abnormality	5	8	9	*n* = 22
Other CNS malformations	13	11	23	*n* = 47
Non‐CNS malformations	*n* = 43 (50.6%)	*n* = 23 (27.1%)	*n* = 19 (22.4%)	*n* = 85 (37.1%)
Face	8	6	4	*n* = 18
Skull	3	4	3	*n* = 10
Heart	17	9	7	*n* = 33
Thorax/diaphragm	1	3	4	*n* = 8
Spine	4	4	3	*n* = 11
Digestive tract	11	5	4	*n* = 20
Urinary tract	9	6	5	*n* = 20
Genitalia	2	4	1	*n* = 7
Extremities	14	4	7	*n* = 25
Other non‐CNS malformations	21	9	7	*n* = 37

*Note:* Data are presented as number of cases (%). Fetuses can have more than one anomaly.

Abbreviations: CNS = central nervous system, VM = ventriculomegaly.

^a^ One way ANOVA *p* < 0.01; post hoc analysis with Bonferroni correction.

^b^ *p* < 0.01 severe versus mild VM.

^c^ *p* < 0.05 moderate versus severe VM.

^d^ *p* < 0.01 moderate versus severe VM.

^e^ *p* < 0.05 moderate versus mild VM.

### 3.2 Outcome

#### 3.2.1 Intrauterine Outcome

Data on the progress of VM was available for 153/229 (66.8%) cases. As shown in Table 3A, VM resolved in 57/153 (37.3%) of cases, predominantly in the mild (36/83; 43.4%), and moderate group (19/43; 44.2%). Progression of VM was shown in 29.4% (45/153) of cases, most often in severe VM (14/27; 51.9%). Seventy‐six cases are described as having unknown outcomes regarding VM evolution, mostly because only one ultrasound was performed at the university medical centers (hospital change). Risk factors for progression, stability, or regression of VM during pregnancy were investigated (Table 3B). Unilateral VM regressed in 45.5% (20/44) of cases and remained stable in another 40.9% of (18/44) cases. Dilatation of the third and fourth ventricles was observed in 54.5% (18/33) and 60% (3/5) of cases associated with progression of VM during pregnancy (*p* < 0.01 between the three different subgroups). In isolated VMs, significantly more cases regressed in atrial width (37/66 cases; 56.1%), whereas in non‐isolated cases more frequently progression was seen (34/87 cases; 39.1%), especially in cases of additional CNS malformations.

**TABLE 3:** Fetal ventriculomegaly: A. Intrauterine outcome, B. Risk factors for regression, stability, and progression during pregnancy.

	Mild VM	Moderate VM	Severe VM	Total
A. Intrauterine outcome	*n* = 83 (54.2%)	*n* = 43 (28.1%)	*n* = 27 (17.6%)	*n* = 153 (100%)
Regressed/resolved	36 (43.4%)	19 (44.2%)^b^	2 (7.4%)^c^	*n* = 57 (37.3%)^a^
Stable	28 (33.7%)	12 (27.9%)	11 (40.7%)	*n* = 51 (33.3%)
Progressed	19 (22.9%)	12 (27.9%)	14 (51.9%)^c^	*n* = 45 (29.4%)^a^
Rate of progression, mean (SD) in mm/week	0.81 (0.57)	2.30 (1.68)	1.84 (2.04)	1.41 (1.53)

	Regression	Stable	Progression	Total
B. Risk factors	*n* = 57 (37.3%)	*n* = 51 (33.3%)	*n* = 45 (29.4%)	*n* = 153 (100%)
Gender				
Male	38 (43.7%)	26 (29.9%)	23 (26.4%)	*n* = 87 (56.9%)
Female	19 (29.7%)	23 (35.9%)	22 (34.4%)	*n* = 64 (41.8%)
Missing	0	2 (100%)	0	*n* = 2 (1.3%)
Unilateral VM	20 (45.5%)^e^	18 (40.9%)	6 (13.6%)	*n* = 44^d^(28.8%)
Bilateral VM	37 (33.9%)	33 (30.3%)	39 (35.8%)	*n* = 109 (71.2%)
Symmetrical	35 (35.7%)	29 (29.6%)	34 (34.7%)	*n* = 98 (64.1%)
Asymmetrical	2 (18.2%)	4 (36.4%)	5 (45.5%)	*n* = 11 (7.2%)
Dilatation of the third ventricle	5 (15.2%)	10 (30.3%)	18 (54.5%)^e^	*n* = 33^d^ (21.6%)
Dilatation of the fourth ventricle	1 (20.0%)	1 (20.0%)	3 (60.0%)	*n* = 5 (3.3%)
Isolated VM	37 (56.1%)	18 (27.3%)^g^	11 (16.7%)^e^	*n* = 66^d^ (43.1%)
Non‐isolated VM	20 (23.0%)	33 (37.9%)	34 (39.1%)	*n* = 87 (56.9%)
Infection during pregnancy	1 (33.3%)	2 (66.7%)	0	*n* = 3 (2.0%)
Fetal cerebral bleeding	0	1 (25.0%)	3 (75.0%)	*n* = 4 (2.6%)
Additional anomalies on ultrasound	19 (23.5%)	30 (37.0%)^h^	31 (38.3%)^f^	*n* = 80^d^ (52.3%)
CNS Malformation	8 (14.8%)	20 (37.0%)^h^	26 (48.1%)^f^	*n* = 54^d^ (35.3%)
Neural tube defect	0	1 (11.1%)	8 (88.9%)	*n* = 9^d^ (5.9%)
Holoprosencephaly	1 (25.0%)	2 (50.0%)	1 (25.0%)	*n* = 4 (2.6%)
Corpus callosum malformation	4 (23.5%)	7 (41.2%)	6 (35.3%)	*n* = 17 (11.1%)
Dandy–Walker continuum	3 (42.9%)	2 (28.6%)	2 (28.6%)	*n* = 7 (4.6%)
Cortical abnormality	0	3 (50.0%)	3 (50.0%)	*n* = 6 (3.9%)
Cerebellar abnormality	2 (16.7%)	2 (16.7%)	8 (66.7%)	*n* = 12 (7.8%)
Non‐CNS malformation	15 (32.6%)	17 (37.0%)	14 (30.4%)	*n* = 46 (30.1%)

*Note:* Number of cases (%). Non‐continuous data are presented as number of cases (%). More anomalies could be assigned to one patient.

Abbreviations: CNS = central nervous system, VM = ventriculomegaly.

^a^ One way ANOVA *p* < 0.05 post hoc analysis with Bonferroni correction.

^b^ *p* < 0.05 moderate versus severe VM.

^c^ *p* < 0.05 severe versus mild VM.

^d^ One way ANOVA *p* < 0.01.

^e^ *p* < 0.05 post hoc analysis with Bonferroni correction: progression versus regression.

^f^ *p* < 0.01; progression versus regression.

^g^ *p* < 0.01; stable versus regression.

^h^ *p* < 0.05 stable versus regression.

Using univariate and multivariate analyses, it was shown that third ventricle dilatation (OR 3.25; 95% CI: 1.89–7.19; *p* = 0.008) and neural tube defects (OR 17.65; 95% CI: 1.71–30.61; *p* = 0.009) were significantly associated with progression of fetal VM. The presence of bilateral VM showed an OR of 2.43 (95% CI: 0.87–7.27; *p* = 0.081) for progression of VM (Supporting Information S1: Table S2).

#### 3.2.2 Overall Obstetric Outcome

Supporting Information S1: Table S1 shows the distribution of live birth, IUFD, and TOP for isolated and non‐isolated VM subdivided into the mild, moderate and severe categories. In 39/229 cases (17.0%), the outcome of pregnancy was unknown, mainly due to lack of information about follow‐up in other hospitals or discharge from follow‐up in case of normalization of the atrial diameter (< 10 mm). The percentage of TOP in the total cohort (including isolated and non‐isolated VM) was 29.3% (24/82 cases) in mild VM, 46.2% (24/52 cases) in moderate VM, and 51.2% (29/56 cases) in severe VM (*p* < 0.05). The percentage of live births was 93.1% (54/58 cases) in mild VM, 78.6% (22/28 cases) in moderate VM, and 92.6% (25/27 cases) in severe VM, after excluding TOP. In the isolated VM group, the percentage of live birth was 100% (38/38 cases) compared to 84% (63/75 cases) in the non‐isolated group (*p* = 0.008). In the non‐isolated VM group, parents decided significantly more frequently for TOP than in the isolated VM group (*p* < 0.05).

The decision for TOP in the isolated group was made in 3.7% (1/27 cases) in mild VM, 27.3% (3/11 cases) in moderate VM, and 42.9% (3/7 cases) in severe VM. The decision for TOP in the non‐isolated group was made in 41.8% (23/55 cases) in mild VM, 51.2% (21/41 cases) in moderate VM, and 53.1% (26/49 cases) in severe VM.

### 3.3 Genetic Evaluation

In total, 143/229 (62.4%) cases opted for genetic analysis; in all cases QF‐PCR was performed, in 129/143 (90.2%) cases CMA, and in 63/143 (44.1%) cases additional SNV‐ES. In 30/76 (39.5%) isolated VM and 113/153 (73.9%) non‐isolated VM, parents opted for genetic analysis (*p* < 0.01). The majority of genetic diagnosis was available before birth/TOP, 11 cases were available just after birth/TOP. In one case with mild VM, genetic testing was performed postnatally due to dysmorphic features after birth, revealing a trisomy 21.

**FIGURE 2:**
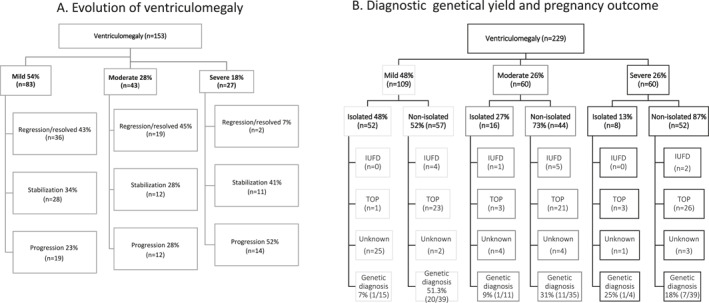
Flowchart for counseling in fetal ventriculomegaly.

A (likely) pathogenic genetic variant was detected in 41/143 (28.7%) cases, of which 21/54 (38.9%) in mild VM, 12/46 (26.1%) in moderate VM, and 8/43 (18.6%) in severe VM (*p* < 0.01 mild vs. severe). One incidental finding was present (a variant in the PIK3R1 gene (c.1425+1G>T p.(?)) in a patient with non‐isolated mild VM). In 3/30 (10%) cases, a genetic diagnosis was established in isolated VM (Table 4A). In the non‐isolated VM group, a genetic variant was found in 38/113 (33.6%) cases (Table 4B). This occurred more frequent (*p* = 0.07) in mild VM (20/39; 51.3%) as compared to severe VM (7/39; 17.9%). In the cases that showed regression of the ventricular diameter during pregnancy, a genetic variant was found in 12.2% (7/57 cases). See Table 5 for details about the (likely) pathogenic variants with genetic analysis. Figure 2 shows a flowchart for counseling in fetal VM regarding evolution of the ventriculomegaly (A), and diagnostic genetic yield and pregnancy outcome (B).

**TABLE 4:** Genetic results of (likely) pathogenic variants in fetal ventriculomegaly.

Total (*n* = 229)	QF‐PCR (*n* = 143)	CMA (*n* = 129)	SNV‐ES (*n* = 63)	Total
Mild VM	9/54 (16.7%)	5/47 (10.6%)	7/19 (36.8%)	21/54 (38.9%)
Moderate VM	3/46 (6.5%)	4/43 (9.3%)	5/21 (23.8%)	12/46 (26.1%)
Severe VM	4/43 (9.3%)	0/39	4/23 (17.4%)	8/43 (18.6%)^b^
Total	16/143 (11.2%)	9/129 (7.0%)	16/63 (25.4%)	41/143 (28.7%)

A. Isolated VM (*n* = 76)	QF‐PCR (*n* = 30)	CMA (*n* = 30)	SNV‐ES (*n* = 9)	Total
Mild VM	0/15 (0%)	0/15 (0%)	1/4 (25%)	1/15 (6.7%)
Moderate VM	1/11 (9.1%)	0/11 (0%)	0/2 (0%)	1/11 (9.1%)
Severe VM	0/4 (0%)	0/4 (0%)	1/3 (33.3%)	1/4 (25%)
Total	1/30 (3.3%)	0/30 (0%)	2/9 (22.2%)	3/30 (10.0%)

B. Non‐isolated VM (*n* = 153)	QF‐PCR (*n* = 113)	CMA (*n* = 99)	SNV‐ES (*n* = 54)	Total
Mild VM	9/39 (23.1%)	5/32 (15.6%)	6/15 (40.0%)	20/39 (51%)
Moderate VM	2/35 (5.7%)	4/32 (12.5%)	5/19 (26.3%)	11/35 (31.4%)
Severe VM	4/39 (10.3%)	0/35 (0%)	3/20 (15%)	7/39 (17.9%)^b^
Total	15/113 (13.3%)	9/99 (9.1%)	14/54 (25.9%)	38/113 (33.6%)^a^

*Note:* Data are presented as number of cases (%).

Abbreviations: CMA = chromosomal micro‐array, QF‐PCR = quantitative‐fluorescence polymerase chain reaction, SNV‐ES = single nucleotide variant analysis using exome sequencing, VM = fetal ventriculomegaly.

^a^ One way ANOVA between VM subgroups *p* < 0.05.

^b^ Post‐hoc analysis with Bonferroni correction: *p* < 0.01 severe versus mild VM.

**TABLE 5:** Overview of (likely) pathogenic findings.

	Diagnosis; #OMIM	Severity VM		
		Mild VM *n* = 21	Moderate VM *n* = 12	Severe VM *n* = 8
**QF‐PCR (*n* = 16)**		***n* = 9**	***n* = 3**	***n* = 4**
Trisomy 13	Patau syndrome	1	0	1
Trisomy 18	Edwards syndrome	2	0	2
Trisomy 21	Down syndrome	4	3	0
Triploidy^a^ ^,^ ^b^		2	0	1

**CMA/karyotyping (*n* = 9)**		***n* = 5**	***n* = 4**	***n* = 0**
arr(22)x3	Trisomy 22	1	0	0
arr[GRCh37] 2p25.3q37.3(205,290‐242,275,551)x2∼3	Mosaicism trisomy 2	1	0	0
arr[GRCh37] 12p13.33q11(173786_37869301)x3∼4 dn	Pallister–Killian syndrome; #601803	1	0	0
arr[GRCh37] 15q14(38575078_38623948)x1 mat	Legius syndrome; SPRED1; #611431	0	1	0
arr[GRCh37] 7p22.3p15.3(43361_23706091)x3,13q33.3q34(107353917_115107734)x1	Unbalanced der(7; 13)	0	1	0
Karyotype: 46,XX,der(13)t(7; 13) (p15.3; q33.3) pat				
arr[GRCh37] 1p36.33p36.32(849467_4131250)x1 dn	1p36 deletion syndrome; #607872	1	0	0
arr[GRCh37] 1q43q44(238466781_249224685)x1 dn	1qter microdeletion syndrome; #612337	1	0	0
arr[GRCh37] 1q43(237511905_237655104)x1 pat	Interstitial deletion 1q	0	1	0
arr[GRCh37] 6q27(170411298_170911237)x1 dn	6q27 deletion syndrome	0	1	0

SNV‐ES/Sanger sequencing/Optical genome mapping (*n* = 16)		*n* = 7	*n* = 5	*n* = 4
CC2D2A; Chr4(GRCh37):g.15575922_15575925dup; NM_001080522.2:c.3744_3747dup; p.(Pro1250fs); heterozygous; maternal	Joubert syndrome type 9; #612285	0	1	0
CC2D2A; Chr4(GRCh37):g.15569105G>C; NM_001080522.2:c.3288G>C; p.(Gln1096His); heterozygous; paternal				
OFD1; ChrX(GRCh37):g.13785278_13785281del; NM_003611.2:c.2632_2635del; p.(Glu878fs); hemizygote	Joubert syndrome type 10; #300804	2	0	0
GLI2; Chr2(GRCh37): c.3462dupA p.(Asp1155Argfs*40); heterozygous; paternal^c^ ^,^ ^d^	Holoprosencephaly type 9; #610829	0	2	0
FGFR3; Chr4(GRCh37):g.1806119G>A; NM_000142.4: c.1138G>A p.(Gly380Arg); heterozygous^d^	Achondroplasia; #100800	0	1	0
ARID1A; Chr1(GRCh37):g.27092946A>G; NM_006015.5:c.2879–2A>G; p.(?); heterozygous, dn	Coffin Siris syndrome; #614607	0	0	1
TUBA1A; Chr12(GRCh37):g.49580448C>T; NM_001270399.1:c.172G>A; p.(Ala58Thr); heterozygous; dn	Lissencephaly type 3; #611603	0	0	1
CCDC88C; Chr14(GRCh37):g.91739503dup; NM_001080414.3:c.5553dup; p.(Ser1852fs); homozygous	Congenital hydrocephalus type 1; #236600	0	0	1
Intronic insertion within intron 2 of SMARCB1; maternal^e^	Atypical teratoid rhabdoid tumor; # 609322	0	1	0
NANS; Chr9(GRCh37):g.100843203C>T; NM_018946.3:c.709C>T p.(Arg237Cys); heterozygous; paternal	Spondylo‐epimetaphyseal dysplasia; #610442	1	0	0
NANS; Chr9(GRCh37):g.100840588T>C; NM_018946.3:c.562T>C p.(Tyr188His); heterozygous; maternal				
COL4A1; Chr13(GRCh37):g.110838820del; NM_001845.5:c.1809del; p.(Pro605fs); heterozygous; paternal^c^	Brain small vessel disease with or without ocular anomalies; #175780	0	0	1
DNAI1; Chr9(GRCh37):g.34459053dup; NM_001281428.1:c.48+2dup; p.?(), homozygous	Primary ciliary dyskinesia; #244400	2	0	0
TET3; Chr2(GRCh37):	Beck–Fahnrer syndrome; #618798	1	0	0
g.74273782C>A:NM_001287491.1:c.738C>A p.(Cys246*) Heterozygous; maternal				
AMPD2; Chr1(GRCh37):	Pontocerebellar hypoplasia type 9 #102771	1	0	0
g.110169050G>C:NM_001257360.1:c.693+1G>C p.(?) Homozygous/biparental				

*Note:* The OFD1 variants were detected in two subsequent pregnancies of the same couple; both children were affected with Joubert syndrome type 10. The GLI2 variants were detected in two subsequent pregnancies of the same couple; both children were affected with holoprosencephaly type 9.

Abbreviations: CMA = chromosomal micro‐array, ES = SNV analysis from exome sequencing, LP = likely pathogenic, P = pathogenic, QF‐PCR = quantitative‐fluorescence polymerase chain reaction, VUS = variant of unknown significance.

^a^ Analysis was performed on DNA isolated from amniotic fluid, expect for post‐mortal biopsy of fibroblasts.

^b^ Chorionic villus biopsy.

^c^ Postnatal blood sample.

^d^ Identified through Sanger sequencing.

^e^ Identified through optical genome mapping; for further details, see reference [8].

## 4 Discussion

This study was conducted to investigate the outcome and risk factors for fetal VM in order to improve prenatal counseling and prognostication. In this retrospective multicenter study in two tertiary referral centers, 229 cases of fetal VM were identified during ultrasound examination, of which 76 (33.2%) cases were isolated. In earlier studies, percentages of 35.6% and 45% of isolated ventriculomegaly were reported, respectively [2, 9].

Primary outcome included the percentage of survival until birth. After excluding TOP and unknown pregnancy outcome, the live birth rate in this study was 93.1% in mild VM, 78.6% in moderate VM and 92.6% in severe VM. A varying range of survival rates of fetal VM has been reported by previous studies. In a study of 194 cases, Lam and Kumar et al. reported a live birth rate of 97.8% in mild VM, 94.4% in moderate VM, and 85.6% in severe VM, including isolated and non‐isolated VM [2]. Fox et al. reported a percentage of live birth of 93%–98% in isolated mild VM, and 80%–97% in isolated moderate VM [10].

In our study, in the isolated VM group, after excluding TOP, the live birth rate was 100% compared to 84% in the non‐isolated group (*p* = 0.008). Robroch et al. reported a percentage of 100% liveborn in the isolated group and 65% in the non‐isolated group [9]. A meta‐analysis by Carta et al. described a percentage of 87.9% of liveborns in isolated severe bilateral VM [1]. The findings of the current study are thus consistent with previous studies regarding live birth rates of isolated and non‐isolated VMs.

In the Netherlands, TOP is legally restricted to the first 24 weeks of gestation, limiting the timeframe available for predicting the evolution of VM during the second trimester. Strength of this study is the availability of longitudinal fetal ultrasound data throughout pregnancy, enabling the identification of risk factors associated with the progression of VM.

In our study, we showed that during pregnancy 43.4% of mild VM cases resolved, of which 75% in isolated VM. In 29.4% of all cases progression of VM occurred. Fetuses with mild and moderate VM remained stable in about 30%, but when severe VM was present, there was a 50% risk of progression. A review by Melchiorre et al. showed stable mild isolated VM in 55.7% of cases, regression in 34%, and progression in 15.7% of the fetuses [5]. Lam and Kumar et al. described a progression rate which increased with severity: in the mild, moderate and severe groups with 1.1, 1.4, and 3.4 mm/week, respectively, with an average progression rate of 1.97 mm/week [2]. With an average progression of 1.41 mm/week (Table 3), our results are comparable with these numbers. Risk factors for progression of VM included 3^rd^ ventricular dilatation at first ultrasound, probably due to aqueductal stenosis and presence of a neural tube defect.

Of the unilateral VM cases, 20 (45.5%) resolved spontaneously, as compared to 37 (33.9%) bilateral cases. These findings support the previously described distinct etiology of unilateral VM, which is characterized by spontaneous normalization of ventricular diameters, potentially attributable to a low grade intraventricular hemorrhage [11]. It was hypothesized that cases diagnosed with unilateral VM may exhibit improved outcomes, which is evident from higher live birth and lower IUFD rates, and less progression of ventricle diameter. This is consistent with our study findings.

VM is frequently associated with underlying genetic disorders. In our study, a genetic diagnosis was established in 28.7% of VM cases in which parents chose to perform genetic analysis; in mild, moderate and severe VMs, this occurred in 38.9%, 26.1%, and 18.6% of cases, respectively. In isolated VM, a genetic variant was identified in 10% of cases. In non‐isolated VM a genetic diagnosis was established in 33.6% of cases. This implies that the presence of associated abnormalities, rather than the severity of VM, is a factor that influences the rate of genetic abnormalities, thus potentially impacting the prognosis. The percentages of genetic abnormalities were higher than those reported in some other studies [12, 13], but in the current study, data of SNV‐ES were also collected. SNV‐ES yielded an additional genetic diagnosis in 27.8% of cases. Thirteen results were available only just after birth/TOP, of which 11 showed pathogenic variants. This could also have influenced our high percentage of genetic diagnoses. In the study by Lam and Kumar et al., a higher percentage of aneuploidy in the mild VM group (overall aneuploidy rate of 14%) was shown. It was concluded that the severity does not predict the risk of genetic anomalies, a finding that was confirmed by Gaglioti et al. [2, 14] A review by Pagani et al. identified 5% abnormal karyotypes in mild isolated VM, and D'Addario et al. found an abnormal karyotype varying from 0% to 26% (mean 2.7%) in isolated mild and moderate VM. The presence of an associated abnormality increased the proportion of genetic disorders to > 15% [12, 13]. In addition to that, Jin et al. stated that deleterious de novo mutations were shown in > 17% of cases of severe ventriculomegaly (including isolated and non‐isolated VM) [15]. More recently, a systemic meta‐analysis showed that after negative CMA in severe bilateral VM, 45% had (likely) pathogenic variants; 54% in non‐isolated cases with additional extracranial anomalies, followed by 38% in non‐isolated cases with additional intracranial anomalies, and in 35% in isolated cases [1]. The findings of our study and recent studies highlight the importance of offering genetic consultations upon the detection of fetal VM, not only in severe cases but also in cases of mild to moderate VM and isolated cases.

There are several limitations to this study. In non‐isolated VM, parents opted more often for genetic analysis (73.9%) than in isolated VM (39.5%), yielding a probable bias in the direction of non‐isolated VM, which could have been a cause of the higher percentage of genetic diagnoses in this group. Furthermore, only 44.1% of SNV‐ES was performed; this could have led to an underestimation of the genetic diagnosis in fetal VM. Secondly, due to the retrospective nature of our study, there were missing data concerning obstetric outcome. A 17% loss to follow‐up (unknown pregnancy outcome) was seen due to normalization of the VM or referral back to the initial caretaker because they did not need ongoing monitoring in the tertiary center. This might also have caused a bias to more severe VM and non‐isolated cases. Third, a notable proportion of pregnancies in the moderate and severe VM groups was terminated. Consequently, it is not feasible to ascertain the natural progression of these pregnancies. Fourth, although this study comprises 229 cases, subgroup analysis was constrained by small cohort sizes. Furthermore, backward selection was used for the Logistic Regression Model. This can lead to models that may not fully reflect theoretical or clinical relevance and may cause an overfit of the data. Since data on the progress of VM was only available for 66.8% of cases, this might have caused a bias in the risk factor analysis for progression, stability, or regression of VM during pregnancy.

Finally, this study identified 70 cases diagnosed after the 24th week of pregnancy. While the legal limit for TOP in the Netherlands is 24 weeks, this might be different in other countries, potentially impacting the generalizability of the findings. For that reason external validation in diverse populations is essential to confirm the generalizability of our results. Future studies should evaluate whether these factors hold in different ethnic groups, low‐resource settings, and varying healthcare environments.

In conclusion, the majority of pregnancies were continued in cases of mild VM, whereas the majority of TOPs were performed in cases of severe VM. In unilateral VM, most children were liveborn. Risk factors for progression of VM during pregnancy were third ventricular dilatation and/or neural tube defects. Overall, genetic abnormalities were present in 28.7% of our total VM cohort. Among those with isolated VM, genetic abnormalities were detected in 7%, 9%, and 25% of mild, moderate, and severe VM cases, respectively. Among those with non‐isolated VM, genetic abnormalities were detected in 5%, 31%, and 18% of mild, moderate, and severe VM cases, respectively. The clinical relevance of genetic analysis was previously recognized, but this study confirms that prenatal testing should be offered to all parents carrying a fetus with VM to aid in prenatal counseling, regardless of VM severity. However, prognosticating fetal VM remains challenging, necessitating repeated imaging. Moreover, larger prospective series incorporating postnatal follow‐up are imperative to enhance parental counseling.

## Conflicts of Interest

The authors declare no conflicts of interest.

## Supporting information

Supporting Information S1

## Data Availability

Data are available on reasonable request.
