# Supplementary material for: Clinical Outcome and Risk Factors for Progression of Prenatally Diagnosed Fetal Ventriculomegaly: A Retrospective Multicenter Study
Source: Prenat Diagn. 2025 May 19;45(9):1089–99. doi: 10.1002/pd.6816 (PMC12322251; doi:10.1002/pd.6816)
Supplement: Supplementary file 1 — Supporting Information S1 [file PD-45-1089-s001.docx]

**Supplemental Material**

**Table 1 SM**: Overall obstetric outcome

|  |  | Live-born | IUFD | TOP | Unknown | Total |
| --- | --- | --- | --- | --- | --- | --- |
| **Total VM** |  | **101 (44.1%)^#^** | **12 (5.2%)** | **77 (33.6%)^%^** | **39 (17.0%)**^†^ | **n=229*** |
| Mild VM |  | **54 (49.5%)** | **4 (3.7%)** | **24 (22.0%)** | **27 (24.8%)** | **n=109** |
|  | Male  Female  Unknown | 32 (51.6%)  22 (48.9%)  0 | 2 (3.2%)  2 (4.4%)  0 | 11 (17.6%)  13 (28.9%)  0 | 17 (27.4%)  8 (17.8%)  2 (100%) | n=62  n=45  n=2 |
|  | Isolated VM  Non-isolated VM | 26 (50.0%)^#^  28 (49.1%) | 0  4 (7.0%) | 1 (1.9%)  23 (40.4%) | 25 (48.1%)^†, &^  2 (3.5%) | n=52*  n=57 |
|  | Unilateral VM  Bilateral VM | 21 (58.3%)  33 (45.2%) | 0  4 (5.5%) | 3 (8.3%)  21 (28.8%) | 12 (33.3%)^†^  15 (20.5%) | n=36*  n=73 |
| Moderate VM |  | **22 (36.7%)** | **6 (10.0%)** | **24 (40.0%)^** | **8 (13.3%)** | **n=60** |
|  | Male  Female  Unknown | 13 (40.6%)  8 (29.6%)  1 (100%) | 4 (12.5%)  2 (7.4%)  0 | 9 (28.1%)  15 (55.6%)  0 | 6 (18.8%)  2 (7.4%)  0 | n=32  n=27  n=1 |
|  | Isolated VM  Non-isolated VM | 8 (53.3%)  14 (31.1%) | 0  6 (13.3%) | 3 (20.0%)  21 (46.7%) | 4 (26.7%)  4 (8.9%) | n=15  n=45 |
|  | Unilateral VM  Bilateral VM | 7 (87.5%)^#^  15 (28.8%) | 0  6 (11.5%) | 1 (12.5%)  23 (44.2%) | 0  8 (15.4%) | n=8*  n=52 |
| Severe VM |  | **25 (41.7%)** | **2 (3.3%)** | **29 (48.3%)^@^** | **4 (6.7%)** | **n=60** |
|  | Male  Female | 12 (44.4%)  13 (39.4%) | 0  2 (6.1%) | 14 (51.9%)  15 (45.5) | 1 (3.7%)  3 (9.1%) | n=27  n=33 |
|  | Isolated VM  Non-isolated VM | 4 (50.0%)  21 (40.4%) | 0  2 (3.8%) | 3 (37.5%)  26 (50.0%) | 1 (12.5%)  3 (5.8%) | n=8  n=52 |
|  | Unilateral VM  Bilateral VM | 3 (50.0%)  22 (40.7%) | 0  2 (3.7%) | 3 (50.0%)  26 (48.1%) | 0  4 (7.4%) | n=6  n=54 |

Data are presented as number of cases (%). Abbreviations: IUD = intra-uterine demise, TOP = termination of pregnancy, VM = ventriculomegaly.

* p<0.05 one way ANOVA between obstetric outcome; post-hoc analysis # p<0.05 live-born versus TOP; & p<0.05 unknown outcome versus live-born; † p<0.05 unknown outcome versus TOP;

^%^ p<0.05 one way ANOVA between VM subgroups; post hoc analysis ^p<0.05 moderate versus mild VM; ^@^p<0.01 severe versus mild VM.

***Table 2 SM.*** *Multiple regression analysis of risk factors for progression of ventriculomegaly*

| Parameters | OR | 95% CI | p-value |
| --- | --- | --- | --- |
| Additional anomalies on ultrasound | 2.89 | 0.49 - 17.11 | 0.242 |
| Bilateral ventriculomegaly | 2.43 | 0.87 - 7.27 | 0.081 |
| Third ventricle dilatation | 3.25 | 1.89 - 7.19 | 0.008 |
| Neural tube defect | 17.65 | 1.71 - 30.61 | 0.009 |
